# Patient with Atrial Myxoma and Signs of Obstruction of the Left Ventricular Outflow Tract

**DOI:** 10.5935/abc.20170007

**Published:** 2017-03

**Authors:** Antonio Fernando Diniz Freire, Alexandre Anderson de Sousa Soares, Tatiana de Carvalho Andreucci Torres Leal, Mucio Tavares de Oliveira Junior, Alexandre de Matos Soeiro

**Affiliations:** Instituto do Coração (InCor) do Hospital das Clínicas da Faculdade de Medicina da Universidade de São Paulo (HCFMUSP), São Paulo, SP - Brazil

**Keywords:** Myxoma/surgery, Hypotension, Tomography, Heart Atria/abnormalities

Female patient, 45 years-old, reported dyspnea on routine efforts with 6 months of evolution with dry cough. Previously healthy she refused to take regular medication. According to the physical examination, she had a heart rate of 105 bpm, blood pressure of 90x60mmHg, respiratory frequency 30 incursions per minute, arterial saturation of 88%, slow capillary filling time, rhythmic sounds, diastolic rumble murmur (+++/6) in mitral focus and bibasal pulmonary crepitations. Chest X-Ray showed a discreet widening of the mediastinum. Chest CT was performed to evaluate the mediastinum and pulmonary parenchyma and an intracardiac mass was found. It evolved with hypotension and worsening of pulmonary congestion.

Emergency echocardiogram showed a moving rounded hyperechoic image in the left atrium, measuring 66x36mm with a pedicle adhered to the membrane of the oval fossa dislocating to the left ventricle (LV) during systole causing hemodynamic repercussion (signs of LV outflow tract obstruction). The patient underwent emergency surgery. Retraction of the tumor revealed a cleft in the anterior cusp of the mitral valve and it was closed. In the immediate postoperative, the patient developed cardiogenic shock refractory to vasoactive drugs; an intra-aortic balloon was implanted, but the patient died 30 hours later. Anatomopathological confirmed the diagnosis of a myxoma: 7.5x4.6x3.4cm. In conclusion, the atrial myxoma with obstruction of the outflow tract of the LV is rare, its clinical manifestations can mislead the evaluator, clinical suspicion and the correct use of propaedeutic are essential for early diagnosis and successful clinical treatment.

**Figure 1:**
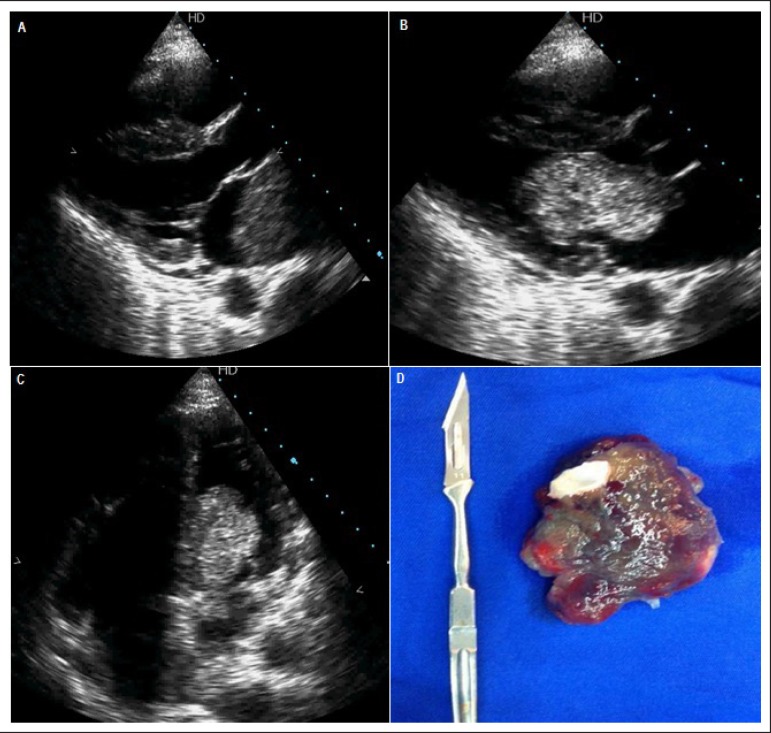
A) Long axis parasternal view in diastole showing hyperechoic image in the interior of the left atrium - round, mobile, with approximately 66x36 mm, with pedicle adhered to the fossa ovalis membrane (myxoma). B) Long axis parasternal view in systole showing atrial myxoma dislocated to the interior of the left ventricle obstructing outflow tract. C) 4-chamber apical view in systole showing atrial myxoma dislocated to the interior of the left ventricle. D) Anatomopathological piece of irregular material of blue-greyish hue, with reddish areas, slightly translucent and sparkly, of gelatinous consistency, measuring 7.5 x 4.6 x 3.4 cm: left atrium myxoma with wide hemorrhage areas.

**Video 1:** Watch the videos here: http://www.arquivosonline.com.br/2017/english/10801/video_ing.asp

